# Anaesthesia Management of Caesarean Section in Two Patients with Eisenmenger's Syndrome

**DOI:** 10.1155/2011/972671

**Published:** 2011-09-26

**Authors:** G. Fang, Y. K. Tian, W. Mei

**Affiliations:** Department of Anaesthesiology, Tongji Hospital, Tongji Medical College, Huazhong University of Science and Technology, Jiefang Ave. 1095#, Wuhan 430030, China

## Abstract

Recently two parturients with Eisenmenger's syndrome underwent caesarean section at our hospital. They were managed by a multidisciplinary team during their perioperative period. The caesarean sections were uneventfully performed, one under general anaesthesia and one with epidural anaesthesia, with delivery of two newborns with satisfactory Apgar scores. One patient died in the post-partum period, and the other did well. We discuss the anaesthetic considerations in managing these high-risk patients.

## 1. Introduction

During a normal pregnancy, parturients undergo dramatic physiological changes in multiple organ systems. Changes in the cardiovascular system include decreased systemic vascular resistance (SVR), increased blood volume, and increased cardiac output (CO) secondary to increased heart rate (HR) and stroke volume (SV). Cardiac disease is a major cause of maternal death, and approximately 25% of maternal cardiac deaths in the last 30 years have been due to congenital heart disease [1]. Eisenmenger's syndrome is classified in the high-risk category, with potentially severe cardiac and neonatal complications [2].

Eisenmenger's syndrome is characterized by right-to-left or bidirectional shunting from severe pulmonary hypertension. Despite advances in medicine, the perioperative maternal mortality remains unacceptably high (estimated at 30%–50%) [3]. The anaesthetic management of caesarean section for parturients with Eisenmenger's syndrome remains an anaesthetic challenge. We present our anaesthetic management of caesarean section of two recent cases. The reporting of these cases was approved by the Institutional Review Board of Tongji Medical College.

## 2. Case Presentation

### 2.1. Case 1

A 19-year-old primigravida (weight 60 kg, height 160 cm) at 33-week gestation was referred to our hospital for VSD with Eisenmenger's syndrome. A cardiac murmur was noted in childhood, but no diagnosis or treatment was performed. She had no symptoms until she developed frequent and severe nausea and vomiting during her pregnancy. Nine days prior to admission, she developed severe fatigue, progressive cough, and shortness of breath. Past medical history was significant only for penicillin allergy. Physical examination revealed cyanosis and clubbing of her fingers. Vital signs were temperature 36.8°C, heart rate (HR) 84 beats·min^−1^, respiratory rate (RR) 24 breaths·min^−1^, blood pressure (BP) 140/95 mmHg, and oxygen saturation by pulse oximetry (S_p_O_2_) 74% on 6 L·min^−1^ of oxygen by facemask. Auscultation revealed a loud P_2_ and a grade 4/6 systolic murmur at the pulmonary area. There were jugular venous distention and mild lower extremity edema. Arterial blood gas analysis on room air demonstrated pH 7.41, P_a_O_2_ 38 mmHg, P_a_CO_2_ 33 mmHg, and S_a_O_2_ 72%. Laboratory tests included hemoglobin (Hb) 13 g·dL^−1^, hematocrit (Hct) 43%, platelets 15 × 10^9^·L^−1^, alanine aminotransferase (ALT) 267 U·L^−1^, aspartate aminotransferase (AST) 230 U·L^−1^, albumin 2.96 g·dL^−1^, D-dimer 1585 ng·mL^−1^, and fibrin degradation products (FDPs) 11.4 *μ*g·mL^−1^. aPTT, PT, electrolytes, and serum creatinine levels were normal. Transthoracic echocardiography showed a 13 mm VSD with prominent right-to-left shunt, dilated right atrium (55 mm) and right ventricle (48 mm), right ventricular hypertrophy (12 mm), moderate-to-severe tricuspid regurgitation, estimated systolic pulmonary artery pressure of 107 mmHg, and an estimated left ventricular ejection fraction (EF) of 74%. Uterine ultrasonography showed IUGR.

The patient was transferred to the intensive care unit (ICU) and treated by a multidisciplinary team of obstetricians, cardiologists, and anaesthesiologists. She received oxygen by facemask with bed rest in the left lateral decubitus position. Dexamethasone 6 mg was given to accelerate fetal lung maturity. In view of her hypoxemic condition and IUGR, a caesarean section was scheduled and metoclopramide and ranitidine were used as aspiration prophylaxis. Upon arrival in the operating room, RR was 21 breaths·min^−1^, HR 99 beats·min^−1^, BP 120/53 mmHg, and S_p_O_2_ 77% on 100% oxygen. General anaesthesia was chosen due to thrombocytopenia. The patient was monitored with electrocardiography, pulse oximetry, and end-tidal capnography, and noninvasive BP and left uterine displacement was applied by a 15° left-tilt of the operation table. As rapid sequence induction (RSI) with predetermined dose of anesthetics may be either excessive or inadequate, RSI was not performed after we weighed the risks of aspiration against hemodynamic instability. Slow induction of general anesthesia with titrate-to-effect etomidate 10 mg was used to avoid dramatic hemodynamic fluctuations. Intubation was facilitated with atracurium 30 mg. Atracurium was chosen to avoid further exacerbation of the compromised liver function. Anaesthesia was maintained with sevoflurane (1-2% end-tidal concentration) in oxygen and remifentanil infusion at the rate of 0.08–0.10 *μ*g·kg^−1^ min^−1^. BP and heart rate were stable (94–123/40–67 mmHg, 79–120 beats·min^−1^) during the 45-minute operation, and S_p_O_2_ remained 67%–76% throughout the uneventful procedure. A female baby was delivered with Apgar scores of 8 at 1 min, 9 at 5 min, and 10 at 10 min. Estimated blood loss was 100 mL, and fluid administration was 550 mL Lactated Ringer's solution. Urine output was 200 mL. The patient was extubated in the operating room, and vital signs in the ICU showed BP 138/88 mmHg, HR 100 beats·min^−1^, RR 26 breaths·min^−1^, and S_p_O_2_ 66%. Blood gas analysis demonstrated P_a_CO_2_ 36 mmHg, P_a_O_2_ 41 mmHg, and S_a_O_2_ 72%. She received transfusion of fresh frozen plasma and platelets in the ICU. Intravenous morphine 3–5 mg was administered by nurse when necessary (nurse-controlled analgesia). The patient was reintubated due to severe hypoxemia 2 hours following extubation. On the 1st and 2nd postoperative days (PODs), her BP and HR were stable with administration of 0.2–0.5 *μ*g·kg^−1^·min^−1^ nitroglycerin, while S_p_O_2_ remained in the range of 62%–74%. On the 3rd POD, her S_p_O_2_ acutely decreased to 42%, and she became unarousable. Despite aggressive resuscitative efforts, she died two hours later. Postmortem examination was denied.

### 2.2. Case 2

A 30-year-old woman (G_2_P_1_, weight 62 kg, height 162 cm) with a 33-week gestation had dizziness, fatigue, dyspnea, and lower extremity edema for 1 week. She was diagnosed with congenital heart disease 10 years ago but remained asymptomatic without therapy until this time. She delivered a female baby by caesarean section without incident at another hospital 6 years ago. There was no other significant past medical history. Vital signs were HR 84 beats·min^−1^, RR 20 breaths·min^−1^, BP 125/80 mmHg, and S_p_O_2_ 58% on room air and 75%–88% on oxygen at 6 L·min^−1^ by facemask. She had marked cyanosis and clubbing of her fingers. Auscultation revealed a loud P_2_ and a grade 5/6 systolic murmur in the pulmonary area. There was moderate lower extremity edema. Laboratory test results included Hb 14.5 g·dL^−1^, Hct 45%, platelets 173 × 10^9^·L^−1^, ALT 271 g·L^−1^, AST 184 g·L^−1^, albumin 3.52 g·dL^−1^, and normal PT and aPTT. Blood gases showed pH 7.35, P_a_O_2_ 61 mmHg, P_a_CO_2_ 34 mmHg, HCO_3_^−^ 18.1 mmol·L^−1^, base excess (BE) −6.6, and S_a_O_2_ 90.1%. Transthoracic echocardiography revealed a 20 mm VSD with bidirectional blood flow at rest, enlarged left ventricle (54 mm), mild dilation of the right atrium, hypertrophy of the right ventricle, mild tricuspid regurgitation, estimated systolic pulmonary artery pressure of 166 mmHg, mild pericardial effusion, and an estimated left ventricular EF of 61%.

After admission to the ICU, she was managed by a multidisciplinary team of obstetricians, cardiologists, and anaesthesiologists. She received oxygen by facemask with bed rest in the left lateral decubitus position. After 3 days of observation, a caesarean section was performed under epidural anesthesia. Left uterine displacement was applied by a 15° left-tilt of the operation table, and a radial arterial line and a central venous catheter were inserted. Initial BP was 149/71 mmHg, central venous pressure (CVP) 10 cm H_2_O, HR 82 beats·min^−1^, and S_p_O_2_ 87%. An epidural catheter was inserted at the L_1_-L_2_ intervertebral space on left lateral decubitus position. Incremental doses of 3–5 mL of 2% lidocaine without epinephrine were administered every 5 min, and a sensory block to the level of T6 was achieved with 21 mL lidocaine over a 30 min period. The sensory blockade level was intendedly kept no higher than T_6_ to avoid significant hemodynamic changes. The surgery proceeded uneventfully without any pain or discomfort. Throughout the procedure, CVP was maintained at 10–12 cmH_2_O. Invasive BP was 150/70 mmHg before delivery, but abruptly decreased to 110/56 mmHg with delivery and responded to norepinephrine infusion at 0.05–0.08 *μ*g·kg^−1^·min^−1^. Blood gas analysis showed P_a_O_2_ 170 mmHg, P_a_CO_2_ 36 mmHg, S_a_O_2_ 99.7%, Hb 12.6 g·dL^−1^, and Hct 38%. Apgar scores were 9 at 1 min and 10 at 5 min. After delivery, tubal ligation was performed. Intraoperative fluid therapy was guided by BP, HR, and CVP, and a total of 400 mL Ringer's solution was administered during the 108-minute surgery. Estimated blood loss was 200 mL. Urine volume was 200 mL. PCEA of 0.2% ropivacaine was started with a background dose of 4 mL·hour^−1^ for analgesia. Epidural opioid was not administered to avoid the adverse effects of respiratory depression, which can be disastrous in these patients. After surgery, the patient was monitored in the ICU for 2 days before transfer to the obstetric ward. Her condition was improving, and S_p_O_2_ was 93% on oxygen at 6 L·min^−1^. On the 5th POD, she had a syncopal episode after walking to the toilet. No other significant postoperative complications occurred. Both the mother and baby were doing well 6 months later.

## 3. Discussion

Pregnancy-induced systemic vasodilation is detrimental in parturients with Eisenmenger's syndrome. Reduced SVR may increase right-to-left shunting and decrease pulmonary blood flow, leading to further hypoxemia with significant risks for both mother and fetus. Anaesthetic management of caesarean section in parturients with Eisenmenger's syndrome requires balancing SVR and pulmonary vascular resistance (PVR).

General anaesthesia is often used for emergency caesarean section. However, positive pressure ventilation may decrease venous return and systemic blood pressure, which can increase the right-to-left shunting. In our hospital, general anaesthesia is used when there are contraindications to neuraxial anaesthesia or in cases of life-threatening emergency with inadequate time for neuraxial anaesthesia. The parturient in our first case had thrombocytopenia. Intravenous etomidate was used for induction to minimize the risks of decreased SVR and cardiac depression. For maintenance of anaesthesia, nitrous oxide was avoided because it is a potent pulmonary vasoconstrictor. Remifentanil can be used to produce stable hemodynamics without subsequent neonatal depression [4]. Maternal mortality remains increased in the first three to four weeks after delivery, so prolonged postoperative care in an intensive care unit setting may be needed [5].

Neuraxial anaesthesia has the advantage of avoiding myocardial depression, but the risk of excessively decreased SVR due to the existence of sympathetic blockade, especially with single-shot spinal anaesthesia. In our second case, small boluses of 2% lidocaine were used to produce a T_6_ block without significant circulatory changes. Epinephrine should be avoided because it can produce tachycardia and arrhythmias, which will increase myocardial oxygen demand and be poorly tolerated in Eisenmenger's syndrome [6]. For patients with Eisenmenger's syndrome, the level of sensory blockade involves a balance between safety and comfort. We kept the sensory blockade level no higher than T_6_, and we think may be it is better than the traditional T_4_ level which can cause dangerous bradycardia with blockade of sympathetic cardioaccelerator fibres in these high-risk patients. A review of 57 articles involving 103 patients showed the safety of regional anaesthesia and recommended its use in Eisenmenger's syndrome [7]. Hemodynamic and respiratory changes are usually minimal with well-managed epidural anaesthesia. However, meta-analysis does not show a significant difference in perioperative mortality between general and regional anaesthesia, and both approaches have significant morbidity and mortality [8]. The choice of general versus epidural-spinal anaesthesia should be made after considering the patient's unique physiology and with consultation with cardiologists, obstetricians, obstetric anaesthesiologists, and cardiac anaesthesiologists.

Vigilant intraoperative monitoring is essential for these patients. Pulse oximetry is the simplest way to assess the degree of right-to-left shunt. Invasive arterial blood pressure and CVP monitoring are recommended. Because blood pressure is often a poor indication of tissue perfusion, monitoring of cardiac output may be needed. The usefulness of a pulmonary artery catheter is controversial in patients with Eisenmenger's syndrome. Transesophageal echocardiography can provide information about cardiac function and intracardiac shunting, but it may not be tolerated in conscious patients. In our second case, continuous arterial and CVP monitoring were valuable in guiding fluid therapy and infusion of vasopressors during operation. 

Titrated perioperative fluid therapy is critical in patients with Eisenmenger's syndrome. Efforts should be made to avoid intravascular hypovolemia from prolonged fasting with consequent dehydration. Sympathectomy-mediated vasodilatation with neuraxial anaesthesia can produce hypotension, so volume expansion and vasopressors should be provided immediately. The use of uterine displacement promotes venous return to the heart. However, excessive fluid administration can overwhelm the compromised right heart. CVP monitoring was indicated in our case to maintain constant filling pressures in the perioperative period.

Management of patients with Eisenmenger's syndrome may include the use of supplemental oxygen, digitalis, diuretics, vasodilators, and anticoagulants. Supplementary oxygen for elective and emergency caesarean section is controversial for healthy patients [9]. In patients with Eisenmenger's syndrome, oxygen is a pulmonary vasodilator which decreases the blood flow across the right-to-left shunt and thereby improves oxygen saturation, so it should be considered for patients with Eisenmenger's syndrome throughout the perioperative period. Maternal arterial oxygen tension should be maintained at 70 mmHg or above when possible. One must be careful in using digitalis with diuretics in patients who are hypoxemic as there is an increased risk of digitalis toxicity. Diuretics may be useful for patients with Eisenmenger's syndrome and severe right heart failure in order to relieve hepatic congestion or increased intravascular volume. This must be done cautiously to avoid decreasing preload below that required to maintain adequate cardiac output with increased right ventricular afterload. Because of possible teratogenicity and adverse effects on uterine circulation, pulmonary vasodilators including prostacycline analogs (Epoprostenol, Treprostinil, Beraprost, and Iloprost), phosphodiesterase inhibitors (Sildenafil, Tadalafil), endothelin receptor antagonists (Bosentan, Sitaxsentan, and Ambrisentan) are not recommended in pregnancy [10]. Use of inhaled nitric oxide during labor has been recommended in Eisenmenger's syndrome. However, it was not available at the time in our hospital. In addition, for patients with severe polycythemia, one needs to consider the danger of increased hyperviscosity, which can lead to stroke and other related complications.

The role of anticoagulation in Eisenmenger's syndrome is controversial. Pregnancy represents a hypercoagulable state, and evidence suggests pulmonary thromboembolism as a cause for maternal demise. However, adverse outcome has been associated with prophylactic heparin therapy in parturients with Eisenmenger's syndrome. Judicious use of antithrombotic drugs and early ambulation may increase survival in patients with Eisenmenger's syndrome. We did not use anticoagulation in the first case because of the high risk of hemorrhage with thrombocytopenia and coagulopathy. The cause of death was discussed by the multidisciplinary team, and pulmonary embolism was a suspected cause of death. Autopsy was suggested to get the definite cause of death, however, it was denied by the family of the patient. 

In summary, patients with Eisenmenger's syndrome undergoing caesarean section present many anaesthetic challenges, but an understanding of the underlying physiology and the use of a multidisciplinary team can guide anaesthetic management.
